# Role of biopsy in diagnosis and treatment of adult celiac disease 

**Published:** 2018

**Authors:** Hugh James Freeman

**Affiliations:** *Department of Medicine (Gastroenterology), University of British Columbia, Vancouver, BC, Canada *

**Keywords:** Celiac disease, Biopsies, Diagnosis

## Abstract

Celiac disease (CD) is an immune-mediated enteropathy that characteristically responds to treatment with a gluten-free diet. In most, clinical features improve with resolution of diarrhea and weight loss. Serological studies also tend to normalize. Small intestinal biopsies from the duodenum reveal a severe to moderately severe architectural disturbance showing crypt epithelial hyperplasia with increased numbers of epithelial cell mitotic figures along with villous “flattening”, increased numbers of lamina propria plasma cells and lymphocytes and increased numbers of intra-epithelial lymphocytes in untreated disease. With a gluten-free diet, these changes can be expected to resolve to normal. In some patients, this mucosal inflammatory process may persist, especially in the proximal small intestine for variable periods of time. In CD, resolution of histopathological changes can occur within 6 months, but often, more than a year is required, and sometimes, 2 years or more. Changes are not only time-dependent, but appear to be gender-dependent with resolution more readily achieved in females compared to males, and age-dependent with more persistence of the inflammatory process in the elderly compared to younger patients. Future studies need to take into account the individual nature of the normal mucosal healing process in CD treated with a gluten-free diet.

## Introduction

 Celiac disease (CD), previously labeled celiac sprue or gluten-sensitive enteropathy, has been defined as an immune-mediated enteropathy that develops in genetically-susceptible persons (1). CD has been characterized as an immune reaction to gluten-containing proteins found in different but related grains, including wheat, rye and barley. Often the disorder presents clinically with diarrhea, malabsorption of one or more nutrients, and resultant weight loss. A mucosal inflammatory response is evident that extends from the duodenum into the more distal small intestine for variable distances. Some believe that the extent and severity of this inflammatory process within the small intestine as well as the timing of its appearance during different stages of adulthood are genetically programmed. 

 In surveys using serological screening methods, it has been estimated that the prevalence rate in most countries approximates 1%. This compares to endoscopic biopsy studies in adults showing higher rates estimated to be about 3% or more (2, 3). In recent years, there has been an increased appreciation that clinical features may also be broad, usually with an array of extra-intestinal features and no or few intestinal symptoms. The ever expanding list of clinical presentations emphasizes that CD is a phenotypically heterogeneous disorder.

 As the clinical recognition of adult CD has increased, some have suggested that there may be a true increase in the disease *per se*. Physician awareness, serological testing and an emergence of CD from obscurity to widespread public interest have clearly played a role. However, other “environmental” factors, may be critically important, including infections that may trigger an inflammatory response to dietary antigens and the development of CD (4) as well as an increasing number of newly marketed medications that may cause mucosal damage, difficult to differentiate from the changes of CD (5, 6). More than ever, it is crucial that initial diagnosis is accurate and subsequent treatment monitored carefully.

## Biopsy for diagnosis of adult celiac disease

 Precise initial diagnosis of adult CD is critical. CD is a life-long disorder and effective treatment with a strict gluten-free diet is difficult and usually very costly and time-consuming for the patient. 

 Two criteria, applied in a sequential fashion, are essential for diagnosis: first, histopathological features of untreated adult CD, the so-called gold standard, should be initially documented; and, second, as this is a gluten-sensitive disorder, convincing evidence of response to a gluten-free diet is critical (1). 

 Historically, serological screening has been used for decades as a means of identifying individuals at potentially increased risk for the disorder. To date, no serological measure has been found to be completely predictive of untreated CD. Serological assessment, however, may be helpful for screening in population studies or even for “case-finding” in clinical practice. However, if CD is clinically suspected (regardless of serological results), a biopsy should be done. 

 In most recent years, antibodies (typically IgA commercial kit tests) to tissue transglutaminase (i.e., tTG) have been popularly used because of their quantitative nature. In contrast, assays focused on endomysial antibodies (i.e., EMA) or many other serologically-based tests are, at best, semi-quantitative, based on “in-house” antigens and difficult to reproduce in different laboratories, or even in the same laboratory using different sources of antigen. Even measurements of antibodies to tTG may pose issues. For example, in genetically at-risk children, spontaneous disappearance of these antibodies has been reported (7). In adults, strongly positive assay results may be present with normal biopsies (8), and some studies have indicated that other disorders may cause falsely elevated results (e.g., chronic liver disease, HIV infection) (9, 10). In contrast, reduced levels may occur in some other clinical settings (e.g., IgA deficiency). Positive serological studies are usually confirmed with biopsies, but in most screening studies, “negative” (or “normal range”) serological results are usually not biopsied to confirm “normal” histologic features. Worse, even with a strong clinical suspicion for CD, some expert clinicians may be dissuaded from biopsy evaluation because of normal levels of antibodies.

 Endoscopic directed biopsy (or even other older methods of biopsy retrieval, e.g., 2- or 4-hole multipurpose, hydraulic) remain exceedingly valuable to obtain critical tissue for definition of the histopathological features suggestive of untreated CD and may be useful for treatment follow-up (11, 12). However, there are many pitfalls to obtaining a quality evaluation.

Endoscopic biopsies tend to be small in size leading some to suggest that only jumbo forceps should be used. In general, however, regular forceps seem sufficient but the biopsies should be gently oriented on paper mesh or filter paper after removal with the mucosal surface outward, rather than simply “shaking” or “swirling” biopsies into a bottle of fixative solution. Individually removed biopsies have been shown to improve quality (compared to the so-called “double-bite” method) and each biopsy or biopsies should be placed in a separate bottle of fixative with the precise site of biopsy defined. Preferably, multiple biopsies should be obtained, including the most proximal duodenal cap region since some believe that this practice may increase overall biopsy detection rate of untreated CD by 10% or more (13). 

 A number of fixatives are available. Formalin is probably most commonly used in most hospitals because of its frequent use for generally larger surgical pathology specimens. Others, however, like Bouin’s or Hollande’s, more rapidly fix small biopsies (compared to formalin), but picric acid (e.g., Bouin’s) may cause unwanted stains of skin and clothing, and may sometimes “leach” granules from some cells, such as eosinophils, potentially leading to difficulty in diagnosis of some disorders (e.g., eosinophilic enteritis). In the laboratory, serial section “ribbons” (usually 4-5 microns for each biopsy) should be taken from the central core of the biopsy to avoid tangential artifact and apparent “pseudo-shortening” of villi. After staining, at least 2 trained specialist observers should review biopsies, ideally to include the endoscopist. This approach, not only improves communication, but usually leads to an improved appreciation of a “positive” or “negative” result and an increased understanding for the degree and extent of architectural and cytological disturbance (particularly, in adult CD) even between biopsies taken at the same endoscopic evaluation.

 A variety of classification methods have been developed over the years in different centers (11, 12, 15, 16). Features of CD are usually most prominent in the proximal small intestine, and in a clinical setting, the most severely abnormal changes are often reported. The degree of altered architecture and other pathological changes may be defined, often in a diffuse but sometimes variable or patchy distribution (11, 12). Biopsy studies along the length of the small intestine show microscopic changes that may extend distally for differing distances, even though early studies with intraluminal tubes into the most distal small bowel demonstrated that even the ileal mucosa is exceedingly sensitive to infused gluten (14). Intra-observer and inter-observer error may occur in interpretation and some disagreement may result in the assessment of the degree or intensity of biopsy change. Interestingly, some have reported that these error factors appear to be even greater with more cumbersome classification methods (16).

 One classification method is focused largely on small bowel mucosal architectural abnormalities (11, 12). Architectural alterations range from severe (or “flat”) changes including absent or rudimentary villi, crypt hyperplasia with an increased mitotic index and an increased mucosal inflammatory cell content in the lamina propria and epithelial compartment (specifically intra-epithelial lymphocyte numbers) to mild changes showing only limited changes in epithelial cell polarity associated with increased numbers of intra-epithelial lymphocytes (or intra-epithelial lymphocytosis). For most adults with untreated disease, severe or moderately severe changes are evident, particularly in the proximal small intestine. In some, mildly abnormal features may be present, often with only intra-epithelial lymphocytosis alone. However, the vast majority (over 80%) with intra-epithelial lymphocytosis alone *do not* appear to have untreated CD (17, 18). Still, some believe that patients with these histopathologic features may be “in evolution” and should be closely followed on a normal diet and, perhaps, re-biopsied at a later date. 

 Clinicians should be wary of a report that indicates biopsies are “diagnostic” for CD. Rather, the initial patient report should read “consistent with untreated CD”. As emphasized above, a response to a gluten-free diet is required to establish the diagnosis. Often, because of potential discomfort and the direct and indirect costs of re-biopsy, a second biopsy is not done and the physician becomes reliant solely on clinical evaluation for follow-up (e.g., resolution of diarrhea, weight gain) and, possibly, a serological assay result that has normalized. Indeed, most patients that ultimately prove to have CD improve with time, despite severely abnormal initial biopsies, as do serological assay results. Although this approach seems practical and reasonable, important pitfalls in diagnosis remain and should be considered.

 First, the characteristic biopsy features of adult CD are not specific or diagnostic. Other causes may result in similar, even identical, biopsy changes (so-called sprue-like intestinal disease). Some include other protein-induced causes of mucosal injury (e.g., soy or milk), infectious agents (such as protozoans, like *Giardia lamblia* and cryptosporidium), tropical sprue syndromes, superimposed nutrient deficiencies (eg., folic acid, vitamin B12, zinc), protein-calorie malnutrition (including extremes, like kwashiorkor), duodenal Crohn’s disease (particularly in the absence of granulomas) and immunoproliferative diseases. Most important, drug-induced sprue-like diseases may occur. Some of these include new pharmacologic drugs (5) as well as novel biological agents (eg., ipilimumab). A particularly noteworthy example is the angiotensin II receptor antagonist, olmesartan (6). This drug, often prescribed for hypertension, may lead to profound diarrhea and weight loss, multiple nutrient deficiencies and small intestinal mucosal biopsies difficult to differentiate from untreated CD. Sometimes, this occurs in an apparently unpredictable fashion after years of drug use. The disorder may be severe and some have required hospitalization. In all of these, removal of the offending cause, or drug, is frequently the keystone to treatment. Only in CD does a response to a gluten-free diet occur. 

 Second, patients with CD previously improved on a gluten-free diet may develop recurrent symptoms and recurrent biopsy changes, so-called “refractory CD”. The usual cause in adults is poor diet compliance, or alternatively, consumption of an unrecognized, often ubiquitous, source of gluten (e.g., pill capsules). Of course, celiac patients may also develop a superimposed cause for symptoms that may alter small bowel architecture, including an infection. Alternatively, the initial diagnosis of CD may have been incorrect or another associated or complicating disorder may have developed (e.g. collagenous sprue, lymphoma). Rarely, some that undergo repeated biopsies do not improve with a strict gluten-free diet. Some of these may have been erroneously labeled with “refractory celiac disease”, however, in most, a response to a gluten-free diet was never initially documented. This unclassified “wastebasket” group of sprue-like small intestinal disease could represent a “resistant” form of CD. Alternatively, others may represent a “difficult to diagnose” or “cryptic” lymphoma. In some, an abnormal subset of intra-epithelial lymphocytes has been described suggesting an important prognostic marker for later lymphoma development (19).

 Finally, serological follow-up may show a quantitative reduction (e.g., tTG units) or even normal results, and yet, numerous studies have confirmed a poor correlation with biopsies (20-23). Often, persistent inflammatory changes are still evident on a gluten-free diet (although normalization eventually occurs). In some respects, serological improvement may be helpful to encourage ongoing patient compliance, but it cannot be relied upon to indicate that mucosal healing has occurred. 

## Biopsy in treatment of adult celiac disease

 Once the disease is suspected and a biopsy confirms changes of untreated CD, treatment with a gluten-free diet is indicated. Resolution of diarrhea should occur accompanied by weight gain. Even in the obese patient recently diagnosed with adult CD, studies have shown that further weight gain may occur. For most, this clinical response, if it occurs within weeks, is usually sufficient to suggest that adult CD is present. In some, this may be accompanied by resolution of an elevated tTG (or other serological test) value. However, as noted above, both clinical resolution and normalization of serological results are not necessarily indicators that the mucosal inflammatory process has resolved.

 In long-term studies with repeated biopsies for follow-up, some patients may respond relatively quickly to a strict gluten-free diet with partial or complete mucosal healing. Remarkably, occasional patients will describe “feeling” subjectively better within days of initiation of diet, followed later by resolution of diarrhea and weight gain. It is conceivable that these early subjective changes of “feelings” are related, in part, to a placebo effect. Resolution of diarrhea and weight gain probably reflect a reduction in the mucosal inflammatory response. Biopsies may improve in many patients, especially females, sometimes completely, within a period of less than 6 months on a gluten-free diet (24). Others may normally require longer time periods on a gluten-free diet to show improvement, sometimes up to 2 years or more. In a recent report (24), up to 90% of patients showed mucosal recovery within 2 years. Regardless of age group, the females in this study responded more readily to a gluten-free diet compared to males. The reason for this sex-based resistance to a gluten-free diet is not known. As well, elderly patients with newly detected changes of untreated CD appeared to be less responsive than younger patients. 

 The histological response in a biopsy from a patient on a strict gluten-free diet takes time to occur. The response includes reappearance of the villi and shortening of less mitotically active crypts. The overall intensity of the inflammatory response, particularly in the lamina propria region, lessens. Sometimes, these changes require longer periods, even 1 to 2 years or more before complete normalization occurs. The reason for the prolonged period that was necessary for recovery in some patients is not known. Possibly, the duration needed for a mucosal response to occur and persist is genetically programmed. In the elderly, this may be due to a longer period on a gluten-containing diet before clinical suspicion CD and the initial biopsy, or simply, greater difficulties in eliminating gluten from the usual diet of an elderly patient. Importantly, information remains limited on temporal changes in mucosal biopsies after use of a strict gluten-free diet in adults with CD.

 Moreover, normalization of abnormal biopsies takes time, especially in the most proximal small intestine in CD (14). Endoscopic biopsies, if repeated in clinical practice, are usually done at similar sites in the proximal duodenum following a prolonged period on a gluten-free diet. In this setting, it is not entirely unexpected that persistent inflammatory changes may be evident. From a clinical perspective, persistent biopsy changes, especially if severe, could lead to mis-diagnosis of refractory disease. Clearly, a thorough exploration of biopsy changes along the length of the small intestine may be required in this clinical setting, particularly if added therapeutic approaches are contemplated.

## Future Considerations

 Duodenal biopsies examined by light microscopy remain “the golden standard” for diagnosis and some believe that electron microscopy may be useful (25). Importantly, new histopathological terms have also been considered in the evaluation of small intestinal mucosal disorders for both diagnosis and treatment. Although current expert histopathological classifications (11, 12, 15, 16) have been devised for clinical and investigative purposes, their precise role continues to be examined, debated and may be evolved. This is reflected in a recent consensus meeting of experts with the emergence of “new” terms, including “microscopic enteritis” (26) and subsequent expert commentary (27). In an era when alternative forms of treatment beyond the gluten-free diet are being considered, precise evaluation in well-defined patients is essential. 

## Conflict of interests

The authors declare that they have no conflict of interest.
